# Nuclear magnetic resonance footprint of Wharton Jelly mesenchymal stem cells death mechanisms and distinctive in‐cell biophysical properties in vitro

**DOI:** 10.1111/jcmm.17178

**Published:** 2022-01-25

**Authors:** Artur T. Krzyżak, Iwona Habina‐Skrzyniarz, Weronika Mazur, Maciej Sułkowski, Marta Kot, Marcin Majka

**Affiliations:** ^1^ Faculty of Geology, Geophysics and Environmental Protection AGH University of Science and Technology Cracow Poland; ^2^ Faculty of Physics and Applied Computer Science AGH University of Science and Technology Cracow Poland; ^3^ Department of Transplantation, Faculty of Medicine, Institute of Pediatrics Jagiellonian University Medical College Cracow Poland

**Keywords:** in vitro characterization, intracellular self‐diffusion, magnetic resonance parameters, mesenchymal stem cells, single‐sided NMR

## Abstract

The importance of the biophysical characterization of mesenchymal stem cells (MSCs) was recently pointed out for supporting the development of MSC‐based therapies. Among others, tracking MSCs in vivo and a quantitative characterization of their regenerative impact by nuclear magnetic resonance (NMR) demands a full description of MSCs’ MR properties. In the work, Wharton Jelly MSCs are characterized in a low magnetic field (LF) in vitro by using different approaches. They encompass various settings: MSCs cultured in a Petri dish and cell suspensions; experiments‐ 1D‐*T*_1_, 1D‐*T*_2_, 1D diffusion, 2D *T*_1_‐*T*_2_ and *D*‐*T*_2_; devices‐ with a bore aperture and single‐sided one. Complex NMR analysis with the aid of random walk simulations allows the determination of MSCs *T*_1_ and *T*_2_ relaxation times, cells and nuclei sizes, self‐diffusion coefficients of the nucleus and cytoplasm. In addition, the influence of a single layer of cells on the effective diffusion coefficient of water is detected with the application of a single‐sided NMR device. It also enables the identification of apoptotic and necrotic cell death and changed diffusional properties of cells suspension caused by compressing forces induced by the subsequent cell layers. The study delivers MSCs‐specific MR parameters that may help tracking MSCs in vivo.

## 1 INTRODUCTION

Mesenchymal stem cells (MSCs) were discovered in the last century by Friedenstein. He observed that bone marrow contains cells that form fibroblast‐like colonies in vitro.^1^ Further studies revealed that MSCs are able to differentiate into different cell lineages namely osteo, chondro and adipo. They can be characterized by the expression of several markers like CD73, CD90 and CD105 and the lack of hematopoietic markers including CD45 and CD34.^2^ Wharton Jelly MSCs, derived from human umbilical cord, represent promising source of stem cells able to differentiate into such cells types as astrocytes, adipocytes, myocytes, cardiomyocytes and neurons.^2^, ^3^

Recently, MSCs attracted considerable attention in the biomedical field as they have been shown to ameliorate symptoms in a number of diseases including neurological and cardiovascular ones.^4^, ^5^ Most studies suggest that MSCs secrete numbers of factors activating the regeneration processes in injured tissues.^6^ For that purpose, MSCs will have to stay alive in the regenerating tissues for a prolonged period of time. However, we are still unable to efficiently trace MSCs in patients after transplantation. Due to the lack of full knowledge about their biology and behaviour after injection, the MSCs cannot be fully utilized in regenerative medicine. Thus, new methods that help to solve these problems are urgently needed. NMR enables the study of porous material or biological systems in a non‐invasive manner and in vitro or in vivo conditions.^7^, ^8^ Tracking the migration of transplanted stem cells with the use of NMR techniques has several years of practice. However, most of the recent research is based on contrast agents labelling the cells.^9^, ^10^, ^11^ *T*_2_ relaxation times or diffusion coefficients, *D*, as biomarkers have only been used in a few papers.^12^, ^13^

The long‐term purpose of the study of live stem cells by means of truly non‐invasive NMR, that is also without contrast agents, is twofold. First of all, it concerns the determination of specific parameters ‘seen’ by low field NMR (LF‐NMR), such as relaxation times *T*_1_, *T*_2_, *T*_1_‐*T*_2_ and *D*‐*T*_2_ maps, or diffusion coefficients, which are characteristic for Wharton Jelly MSCs. The proposed multi‐parametric characterization is also implemented to obtain a set of MR parameters in order to minimize the possibility of overlapping signals from other cells. These parameters may be useful in‐cell detection when studying animal models or patients by means of MRI in vivo. A similar approach was developed and implemented for porous and heterogeneous systems.^14^, ^15^ Secondly, NMR parameters characterizing in vitro cell suspensions can be used to determine their quantitative and qualitative characteristics, such as size, self‐diffusion coefficient and viability. For this purpose, besides results from the characterization of MSCs by LF‐NMR, a single‐sided Mobile Universal Surface Explorer (MoUSE) was used. MoUSE allows the study of a sample using an extremely strong magnetic field gradient (~24 T m^−1^) and short diffusion times, which leads to higher diffusion weighting without coming into motional averaging between compartments. New promising cell studies, carried out under these conditions and considering several signal components from cell samples, have appeared recently.^16^, ^17^ Another advantage is the ability to test samples in open geometry with the use of mobile apparatus,^8^ which increases the potential of future uses in the case of finding optimal measurement protocols and parameters dependent on cells characteristics.

## 2 MATERIALS AND METHODS

### 2.1 Experimental model

The umbilical cords were collected after Caesarean sections. Written consents were obtained from parents. The umbilical cords were washed with phosphate‐buffered saline supplemented with antibiotic‐antimycotic solution, cut into small explants and plated into a plastic flask. Explants were cultured with a growth medium for MSCs (DMEM Low Glucose, Biowest), supplemented with the platelet lysate in standard culture conditions under 21% of O_2_ and 5% of CO_2_ at 37°C. Next, the explants were removed, and the cells were passaged using the Accutase cell detachment solution (BioLegend). After reaching the appropriate number of cells, WJMSCs were used for further experiments.

### 2.2 WJMSCs characterization

The phenotype of WJMSCs was analysed according to the International Society of Cellular Therapy standards. Briefly, cells cultured at passage 3 or 4 were collected and stained with antibodies against CD73, CD90, CD105, CD3, CD45, CD34, CD14 and CD19 (Becton Dickinson) for 30 min at 4°C in darkness. Appropriate isotype controls were used to exclude non‐specific binding. Cells were analysed using Attune Nxt Flow cytometer (Thermo Fisher Scientific, Waltham, MA, USA), and data were analysed using Attune NxT Software v2.2.

WJMSCs were tested for their three‐lineage differentiation potential using MesenCult Adipogenic Differentiation Medium, MesenCult Osteogenic Differentiation and MesenCult ACF Chondrogenic Differentiation Medium (all from StemCell Technologies, Vancouver, CA‐BC, Canada). For analysis, cells were seeded into 12‐well plate at a density of 1.3 × 10^3^ cells/cm^2^ and cultured in the standard medium until the culture reached appropriate confluence and the medium was replaced by differentiation medium. At the end of differentiation, cell was stained with Oil Red O (adipocytes) (Sigma‐Aldrich) Alizarin Red (MERC) (osteoblasts) and Alcian Blue staining (chondrocytes) (Sigma‐Aldrich) according to standard procedures.

### 2.3 Experiments in a LF‐NMR system with a bore aperture

A suspension of MSCs from Wharton Jelly in a PBS buffer was put into glass pipette and centrifuged. Then, the glass pipette was closed and so the prepared samples were examined on a Magritek Rock Core Analyzer at a magnetic field of 0.05 T. Samples with 5 and 15 million cells in a volume of 0.5–1 ml were tested (suspensions a–d, see Table 1). The Inversion Recovery (IR) and Carr‐Purcell‐Meiboom‐Gill (CPMG) sequences were used for 1D‐*T*_1_ (inter‐experiment delay, ID = 5 s, *T*_1_ delay range: 0.1–5 s) and *T*_2_ (ID = 7.5 s, echo time, TE = 200–400 μs, number of echoes in CPMG encoding train, NoE = 50,000) measurements, respectively. 2D *T*_1_‐*T*_2_ correlation maps were obtained with IR‐CPMG sequence ID = 3 s for buffer, ID = 350 ms for cells, *T*_1_ delay range: 0.1–5 s, TE = 400 μs, NoE = 20,000). In order to enhance the signal from cells, shorter inter‐experiment delays were applied for *T*_1_‐*T*_2_ (350 ms) than in the case of 1D experiments. For 2D complementary diffusion experiments, a diffusion‐weighted pulsed‐field gradient spin‐echo (PGSE) sequence was applied with an increasing gradient amplitude to 0.5 T m^−1^ and CPMG sequence for detection (ID = 350 ms, TE = 400 μs, NoE = 10,000, gradient pulse length, *δ* = 6 ms for suspensions a and c, *δ* = 8 ms for suspension d, interval between two gradient pulses, *Δ* = 20 ms). The maximum *b*‐value achieved for suspensions a and c was equal to 11.6 × 10^9^ sm^−2^ and 19.8 × 10^9^ s m^−2^ for suspension d. All the experiments were conducted in seven separate experimental series, and for each cell concentration measurement with the same parameters was repeated at least once. In the work, representative data were shown.

**TABLE 1:** Peak positions from 1D‐*T*_1_, *T*_2_ distributions (A) and 2D *T*_1_‐*T*_2_ (B) and *D*‐*T*_2_ (C) correlation maps

A. Results from 1D‐*T*_1_ and *T*_2_ distributions						
Sample	MSC: *V*_tot_ [no of cells: ml]	*T*_2_ [ms] Peak 1	*T*_2_ [ms] Peak 2	*T*_2_, _log‐mean_ [ms]	*T*_1_ [ms]	*T*_1, log‐mean_ [ms]
Buffer (a)	0: 1	2584.2	–	2640.3	2439.6	2479.6
Cells (b)	5: 1	2770.0	117.6	2857.8	2158.0	2108.2
Cells (c)	5: 0.5	2584.2	227.5	1846.3	2294.0	2166.4
Cells (d)	15: 0.5	4201.2; 1162.3	310.8	1884.6	1795.3	1815.6

B. Results from *T*_1_‐*T*_2_ correlation maps									
Sample	MSC: *V*_tot_ [no of cells: ml]	*T*_1_ [ms] Peak 1	*T*_2_ [ms] Peak 1	*T*_1_ [ms] Peak 2	*T*_2_ [ms] Peak 2	*T*_1_ [ms] Peak 3	*T*_2_ [ms] Peak 3	*T*_1_/*T*_2_ Peak 1	*T*_1_/*T*_2_ Peak 2
Buffer (a)	0: 1	2740	2740	–	–	–	–	1	–
Cells (c)	5: 0.5	3090	3310	923	240	–	–	0.93	3.8
Cells (d)	15: 0.5	3490	3145	1120	333	–	–	1.11	3.4

C. Results from *D*‐*T*_2_ correlation maps									
Sample	MSC: *V*_tot_ [no of cells: ml]	*D*_1_ [×10^9^ m^2^/s] Peak 1	*T*_2_ [ms] Peak 1	*D*_2_ [×10^9^ m^2^/s] Peak 2	*T*_2_ [ms] Peak 2	*D*_3_ [×10^9^ m^2^/s] Peak 3	*T*_2_ [ms] Peak 3	*D*_1_/*D*_2_	*D*_1_/*D*_3_
Buffer (a)	0: 1	2.08	2300	–	–	–	–	–	–
Cells (c)	5: 0.5	2.04	2340	0.93	185	0.163	238	2.19	12.55
Cells (d)	15: 0.5	2.19	2880	1.45	240	0.216	266	1.51	10.14

### 2.4 Experiments in a LF‐NMR single‐sided system

For the NMR measurements in a constant time‐steady gradient, a single‐sided MoUSE scanner (NMR‐MoUSE, Magritek) was used with a magnetic field, *B*_0_ of 0.5 T and constant time‐steady magnetic field gradient of 24 T m^−1^ (1030 MHz mm^−1^) set perpendicularly to *B*_0_ and longitudinally to slice thickness. A profile sequence was used to localize the bottom of the Petri dish (repetition time, RT = 2 s, TE = 128.5 μs, *Δ* = 10 ms, number of echoes in CPMG encoding train, NoE = 512, slice thickness, ST = 10 μm) or cylindrical container (RT = 6.2 s, TE = 50.5 μs, Δ = 20 ms, NoE = 4098, ST = 20 μm) and the presence of the examined material. The precise lift in the MoUSE device allowed us to set the position of the slices. Echo decays with tau from 0.01 to 0.05 ms for Petri dishes, which corresponded to *b*‐values from 0.04 to 1.03 × 10^9^ s m^−2^ and 0.01 to 0.2 ms (*b*‐value in the range of 0.08–33.1 × 10^9^ s m^−2^) for cylindrical container were registered. Then, the obtained data were calculated using the Inverse Laplace Transform (ILT) (L&H algorithm, Prospa software) and fitted independently using a one‐ or bi‐exponential model.

### 2.5 Quantification and statistical analysis

The registered data were analysed using ILT with Lawson&Hanson and FISTA algorithms,^18^ allowing us to obtain 1D distributions and 2D maps, respectively (Prospa software, Magritek). Data from single‐sided NMR‐MoUSE were additionally processed by fitting independently a mono‐ or bi‐exponential diffusion model (for descriptions please see for example in the work of Mazur and Krzyżak^16^) in Statistica (TIBCO Software Inc.).

## 3 RESULTS

### 3.1 WJMSCs characterization

**FIGURE 1:**
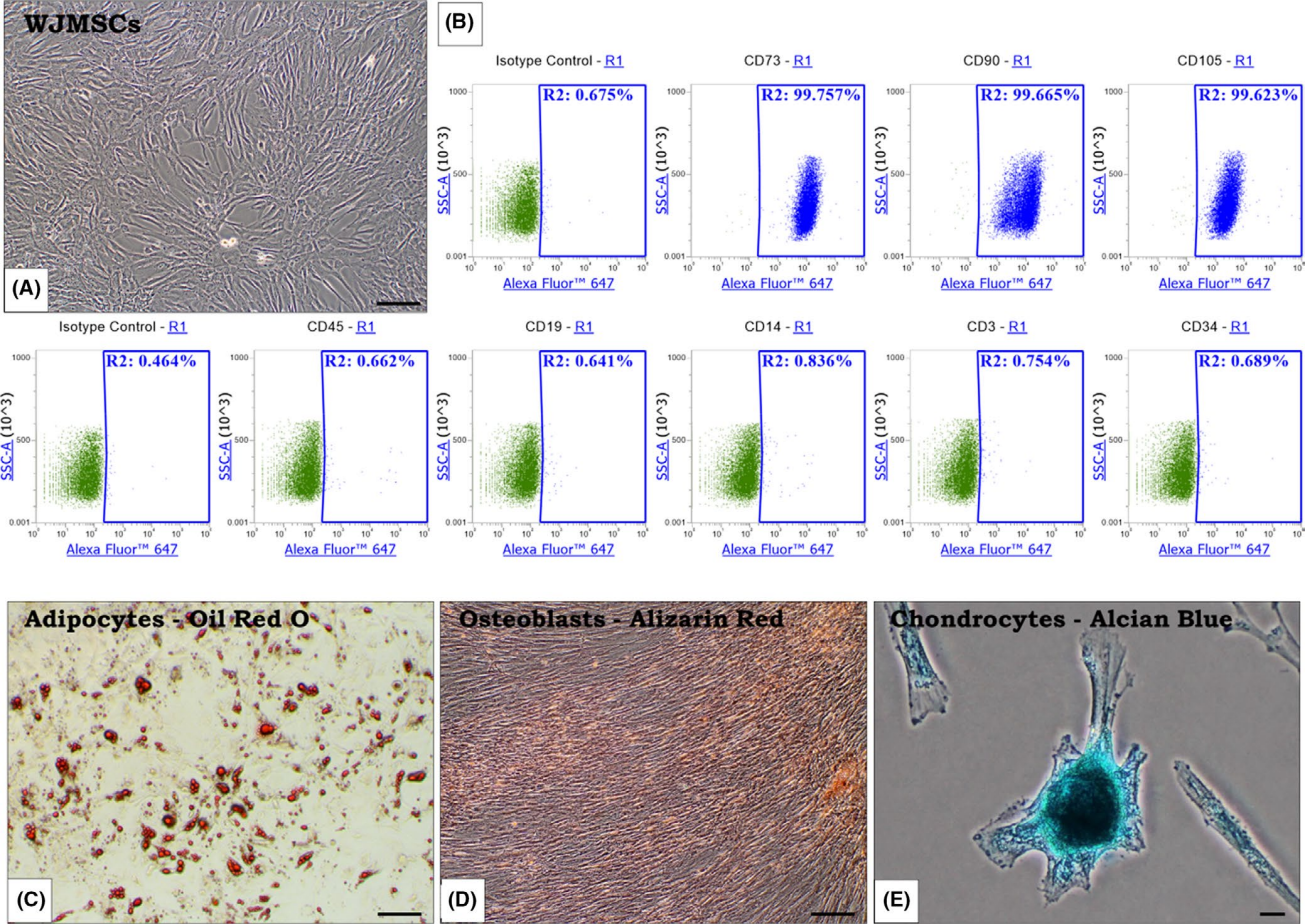
Characterization of WJMSCs in standard culture conditions; light microscopy, magnification ×100, bar =100 µm (A). The expression of surface markers characteristic for MSCs: CD73, CD90 and CD105 (over 90% positive cells). The cells are negative for hematopoietic antigens: CD45, CD19, CD14, CD34, CD3; flow cytometry analysis (B). Trillineage differentiation potential of WJMSCs: adipocytes (Oil Red O staining) (C), osteoblasts (Alizarin Red s staining) (D) and chondrocytes (Alcian blue staining) (E); light microscopy, magnification ×100, bar =100 µm

The WJMSCs show the minimal criteria outlined for MSCs by the International Society of Cellular Therapy. They adhere to plastic surface in standard culture conditions and display fibroblast‐like morphology (Figure 1A). Cytometric analysis revealed high expression of specific mesenchymal markers. More than 90% of cells were CD73, CD90 and CD105 positive, whereas they do not express hematopoietic antigens (CD45, CD14, CD19, CD34 and CD3) (Figure 1B). We have also confirmed multipotent differentiation potential of WJMSCs. These cells demonstrated strong capacities for differentiation towards adipogenic (Figure 1C), osteogenic (Figure 1D) and chondrogenic (Figure 1E) lineages.

### 3.2 *T*_1_ and *T*_2_ relaxation

**FIGURE 2:**
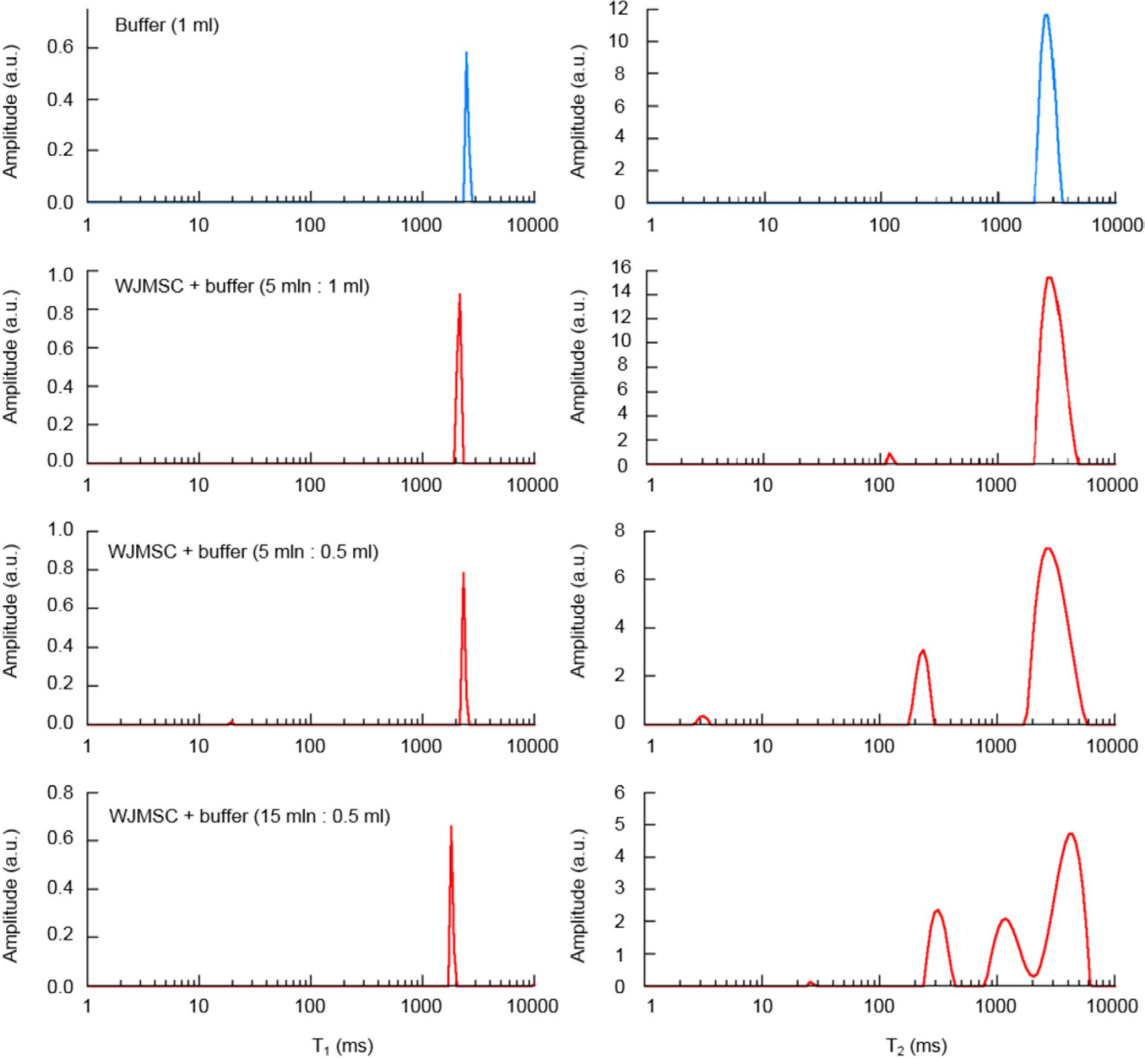
Relaxation times 1D distributions. *T*_1_ (left panel) and *T*_2_ (right panel) relaxation times distributions obtained for different cells concentration in the suspensions

In Figure 2, the *T*_1_ and *T*_2_ distributions for MSCs samples with various amounts of cells in a specified volume are presented, and in Table 1, the relaxation times at maximum and *T*_1,2_ log‐mean values are collected. In the case of T_1_ distributions only one peak is visible, for both the buffer and for the cell samples (see Figure 2, left panel), having *T*_1_ from 2.16 to 1.8 s for suspensions b to d, respectively. The lack of a clearly separated peak derived from the cells is probably caused by the close values of *T*_1_ for buffer and cells samples, which makes it difficult to separate these two components using ILT.

On *T*_2_ distributions (Figure 2 right panel), a separate peak for MSCs can be seen even for the lowest cells concentration. *T*_2_ of MSCs was equal to 118, 228 and 311 ms for suspensions b, c and d, respectively (the difference is caused by the effect of different amount of MSCs signal on ILT). Suspension d probably contained cells clusters with intercellular spaces resulting in additional component with *T*_2_ = 1162 ms.

### 3.3 *T*_1_‐*T*_2_ and *D*‐*T*_2_ correlation maps

**FIGURE 3:**
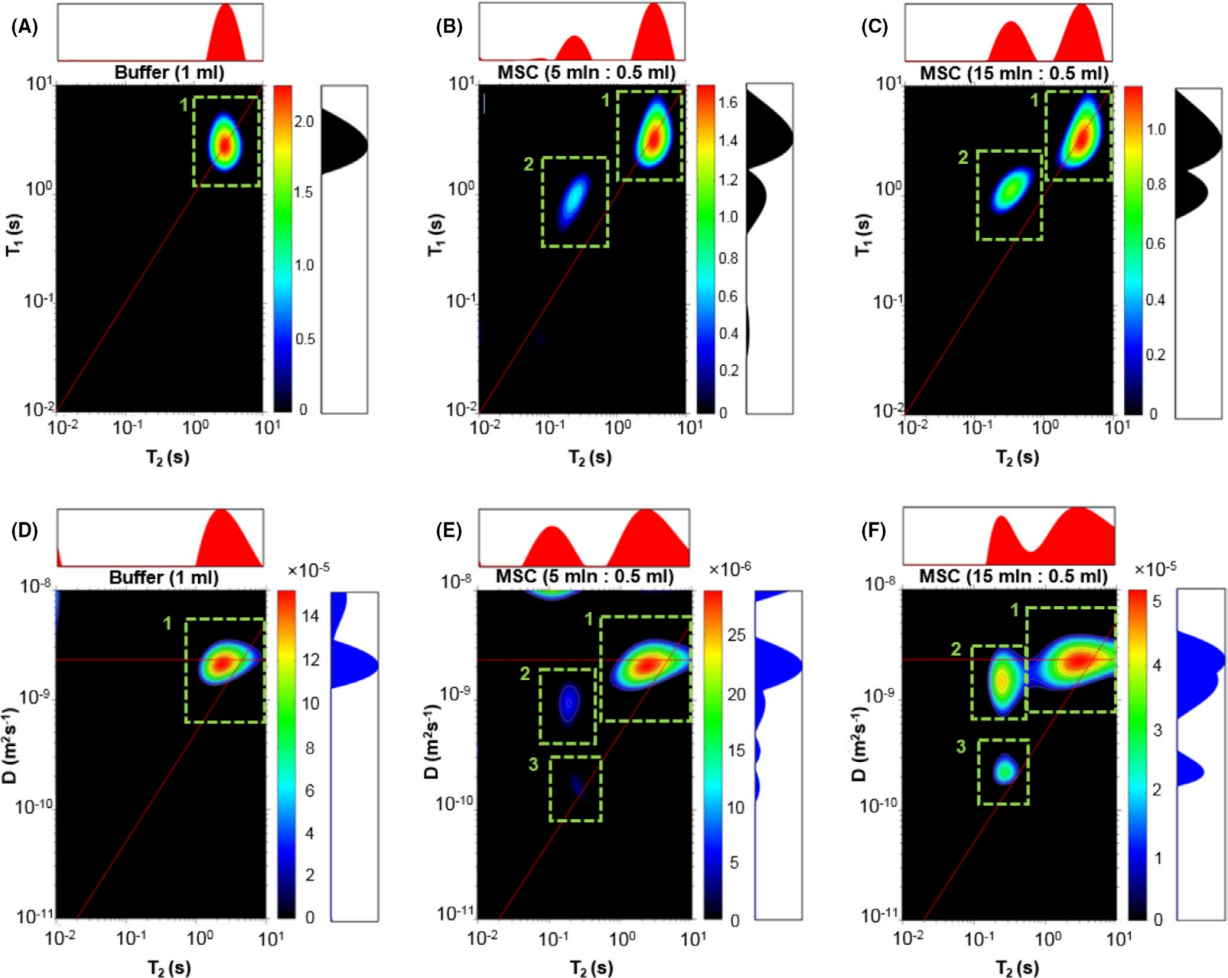
Correlation maps from 2D experiments. *T*_1_‐*T*_2_ correlation maps (A–C) and *D*‐*T*_2_ correlation maps (D–F) for buffer and MSCs samples with peaks numbered from 1 to 3

In Figure 3A–C, *T*_1_‐*T*_2_ maps are presented corresponding to the 1D distributions from Figure 2 for suspension a, c and d. A peak with an increasing intensity and area for the cell samples, located at *T*_2_ about 130–350 ms and not present for the pure buffer sample, is the main observation for these measurements. Its *T*_1_/*T*_2_ values were a few times higher than for a free water, which is another confirmation of the assumption that the signal originates from the restricted region of the sample.

A comparison of *D*‐*T*_2_ maps for the pure buffer and MSCs samples is shown in Figure 3D–F. It can be observed that for the used PGSE parameters signal with *T*_2_ from the range of 130–350 ms was separated into two components with different diffusion coefficients, which was the most visible for the suspension d. For this concentration, the first component (Figure 3F) is characterized by diffusion coefficient of 1.45 × 10^−9^ m^2^ s^−1^, and the latter: 0.216 × 10^−9^ m^2^ s^−1^, which is 1.5 and 10.1 lower than the diffusion coefficient for the main peak, originating from free water within this sample. For the lower concentration (suspension c), the corresponding components have values of 0.93 × 10^−9^ m^2^ s^−1^ and 0.163 × 10^−9^ m^2^ s^−1^, respectively. The lowest diffusion may be related to the water compartment with the highest restriction—probably intracellular spaces, while the second component might originate from the restricted areas between cells, and may be the same as the signal at 1162 ms of 1D‐*T*_2_ distribution.

### 3.4 Diffusion measurements of cells cultured in a Petri dish

**FIGURE 4:**
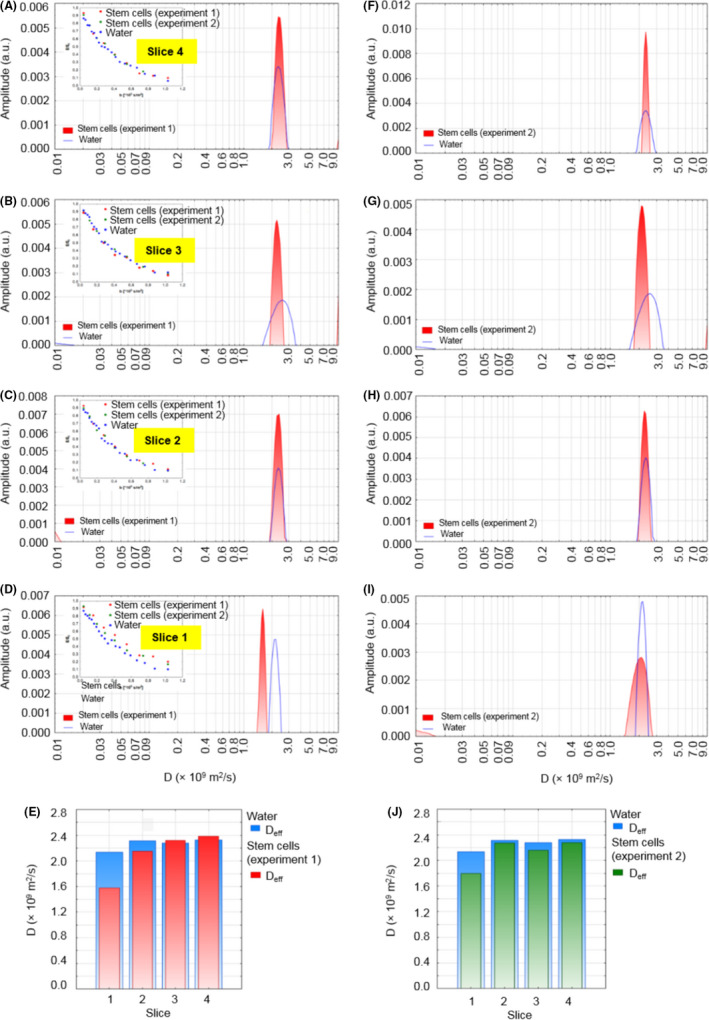
Two independent diffusion experiments in Petri dishes. Data in the left column represent experiment 1, while in the right column: experiment 2, conducted independently. Diffusion coefficient distributions for water and cells samples in Petri dishes, sequentially for the layers from the highest (‘4’) to the lowest (‘1’) (A–D, F–I). Inset graphs present attenuation of signals, *E*/*E*_0_, vs. *b*‐value. Column plots comparing diffusion coefficients *D*_eff_ for cells and water sample (E), (J)

In Figure 4A–D and F–I, diffusion distributions obtained for samples of stem cells cultured on Petri dishes are shown and compared with the results of pure water examined under the same conditions. Four slices of 10 µm were registered for two samples prepared independently—bottom slices are assigned as ‘1’ and top slices as ‘4’. Simultaneously, effective (ie averaged for all of the water pools) diffusion coefficients were fitted using a mono‐exponential function (results shown in Table 2) and presented in Figure 4E and J. Effective diffusion coefficients for the bottom slices of the stem cell samples (slice 1, Figure 4D and I) were 1.2–1.5 times lower than values of coefficients obtained for slices situated above them (slices 2–4). Diffusion coefficients for slice ‘1’ for cells were also 1.2–1.5 times lower than for each water measurement. Results for slices 2–4 are close to the water diffusion coefficients.

**TABLE 2:** Results of fitting of mono‐exponential functions for stem cells and water sample in a Petri dish for slices of 10 µm (A) and bi‐exponential functions for stem cells and mono‐exponential for water sample in a cylindrical container for slices of 50 µm (B)

A. Petri dish				
Slice number	Slice width [µm]	Water	Stem cells (experiment 1)	Stem cells (experiment 2)
		*D*_eff_ [×10^9^ m^2^/s]	*D*_eff_ [×10^9^ m^2^/s]	*D*_eff_ [×10^9^ m^2^/s]
1 (bottom)	10	2.13 ± 0.06	1.58 ± 0.07	1.80 ± 0.04
2	10	2.31 ± 0.07	2.15 ± 0.06	2.27 ± 0.08
3	10	2.28 ± 0.06	2.32 ± 0.09	2.16 ± 0.05
4 (top)	10	2.32 ± 0.05	2.38 ± 0.10	2.28 ± 0.08

B. Cylindrical container						
Slice number	Slice width [µm]	Water	Stem cells	Stem cells	Stem cells after 6 days	Stem cells after 6 days
		*D* [×10^9^ m^2^/s]	*D*_1_ [×10^9^ m^2^/s]	*D*_2_ [×10^9^ m^2^/s]	*D*_1_ [×10^9^ m^2^/s]	*D*_2_ [×10^9^ m^2^/s]
1 (bottom)	50	2.31 ± 0.003	1.36 ± 0.07	0.068 ± 0.026	0.46 ± 0.09	0.042 ± 0.014
2	50	2.47 ± 0.05	1.19 ± 0.07	0.052 ± 0.027	0.65 ± 0.06	0.082 ± 0.015
3	50	2.50 ± 0.03	1.16 ± 0.08	0.064 ± 0.030	0.56 ± 0.03	0.031 ± 0.015
4 (top)	50	2.48 ± 0.03	1.14 ± 0.06	0.055 ± 0.026	0.78 ± 0.05	0.084 ± 0.023
Mean	50	2.44 ± 0.03	1.21 ± 0.07	0.0598 ± 0.027	0.61 ± 0.06	0.0598 ± 0.017

### 3.5 Diffusion measurements of cell suspension in a cylindrical container

**FIGURE 5:**
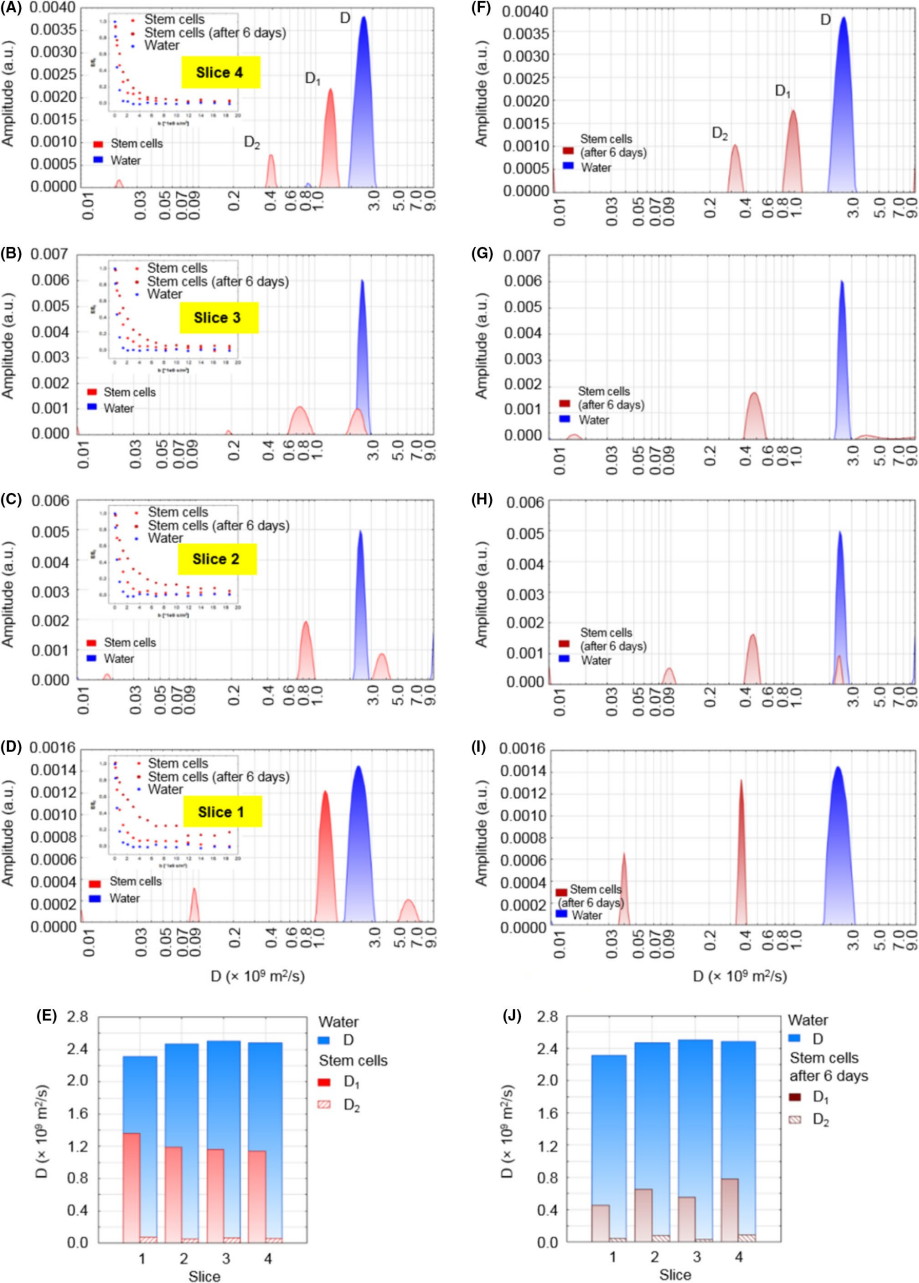
Diffusion experiments in cylindrical container. Data in the left column represent samples measured immediately after preparation, while in the right column: after 6 days. Diffusion coefficient distributions for water and cells samples within a cylindrical container, sequentially for the layers from the highest (‘4’) to the lowest (‘1’) (A–D, F–I). Inset graphs present the attenuation of signals, *E*/*E*_0_, vs. *b*‐value. Column plots comparing diffusion coefficients *D*_1_ and *D*_2_ for cells and *D* for water samples (E), (J)

In order to obtain the results of diffusion coefficients for cells less biased by water present between them, samples of a centrifuged cell suspension were examined in a cylindrical container. Distributions of diffusion coefficient for four slices with width of 50 µm are compared in Figure 5A–D. Results of fitted values using bi‐exponential model are listed in Table 2 and visualized in Figure 5E. Measurements for these samples were also repeated after 6 days (see Figures 2J and 5F–I).

For samples measured immediately after preparation, significantly lower, 1.7–2.2 times, diffusion coefficients *D*_1_ for all the examined slices than the corresponding values for water can be noticed. Values from 1.36 to 1.14 × 10^−9^ m^2^ s^−1^ were registered, in comparison with 2.31–2.48 × 10^−9^ m^2^ s^−1^ obtained for water. Moreover, second diffusion component with *D*_2_ ranging between 0.052 and 0.068 × 10^−9^ m^2^ s^−1^ was possible to obtain. For samples examined after 6 days of incubation at room temperature, a significant decrease of diffusion coefficients was observed (1.5–3 times). The lowest values of the diffusion coefficient for the bottom slice and generally increasing values for higher located slices can be noticed.

## 4 DISCUSSION

In the work, different NMR approaches were applied in order to characterize MSCs. Each of them delivered distinct information which was complementary to the others, and all are discussed below.

### 4.1 Determination of the MSCs’ size

Mesenchymal stem cells’ diameter ranges from *d*_min_ = 15 μm to *d*_max_ = 30 μm.^19^ For these values and real suspension volumes, theoretical cellular fractions were calculated and compared with the ones from *D*‐*T*_2_ experiment (Figure 3). Fractions coincide for *d*_min_ =15 μm, which is assumed to be real MSCs size. Note that MSCs size cannot be determined from *T*_1_‐*T*_2_ maps, because extra‐ and intracellular water can have very similar relaxation times^16^ and they may combine into a single peak disenabling fractions comparison.

### 4.2 The influence of MSCs cultured in a Petri dish on an apparent diffusion coefficient

Mesenchymal stem cells cultured on a Petri dish were traced by applying a very small slice thickness, which was possible due to the use of a single‐sided NMR‐MoUSE device. The slice thickness of 10 μm ensured a single layer of cells to be examined in a single slice. Due to the considerable reduction of the effective diffusion coefficient in the bottom slice, the presence of a significant number of cells is suspected. Results for slices 2–4 are similar to water diffusion coefficients, suggesting that the diffusion coefficient is only affected by the presence of cells in the first and the lowest layer on the bottom of the Petri dish. Using *D*_cyto_(*t*_d_ = 10 ms) from simulations (see Section 4.), cells fraction on the bottom of a Petri dish can be estimated to be in the range of 23–34%. Hence, cells did not completely cover the surface and the proportion of water between the cells strongly influenced the detection of a true intracellular self‐diffusion coefficient. However, these findings seem useful for understanding the impact of MSCs on the apparent (ie dependent on diffusion time) diffusion coefficient of water in vivo measured in a clinical practice. The effective diffusion coefficient may reflect the amount of MCSs accumulating on tissue after a medical intervention.

### 4.3 Monitoring of diffusion and viability of MSCs cultured in a cylindrical container

Values of *D*_1_ for samples examined in the cylindrical container are much lower than those measured in Petri dishes. This is probably because of a lower proportion of water between cells in the samples prepared in this way. This may suggest obtaining closer values of effective diffusion coefficient to the true self‐diffusion intracellular coefficients in MSCs. The second diffusion component may originate from structures located within the cells which cause greater water restriction. Immediately after the preparation of the cell suspension, the dependence of the diffusion coefficient on the slice location is rather random and they can be averaged in order to obtain more reliable values. Mean diffusion coefficients can be used for the identification of the second component, *D*_2_.

#### 4.3.1 Evidence of diffusion in the in‐cell structures

The MSCs nucleus is a large and round cellular structure^20^ and was suspected in the first place to contribute to the second diffusion component. For example, in yeast, nuclear to cellular volume ratio is equal to about 8%,^21^ which is associated with a nuclear radius of ~1 μm.^22^ In the mouse MSCs, the ratio of cell and nucleus diameters was reported to be equal to about 63%.^23^ In human MSCs, the ratio of nuclear to cellular diameters is equal to 26–31%.^24^ Assuming the cell radius of 7.5 μm estimated in Section 1, nucleus radius, *R*_nucl_, is equal to ~2 μm. This size and *D*_0,nucl_ = 0.095 × 10^−9^ m^2^ s^−1^ reported by Mazur and Krzyżak^16^ yielded *D*_nucl_(*t*_d_ = 20 ms) = 0.0602 × 10^−9^ m^2^ s^−1^, which is in a good agreement with the mean value of *D*_2_ equal to 0.06 × 10^−9^ m^2^ s^−1^. Therefore, it is highly possible that nuclei in the examined cells have a radius of *R*_nucl_ = 2 μm.

#### 4.3.2 Cell viability reflected in the effective diffusion coefficient

For samples examined after 6 days of incubation at room temperature, a significant decrease of diffusion coefficients was observed (1.5–3 times). This may be related to structural damage to the cells over time, as well as with partial water evaporation. The lowest values of diffusion coefficient for the bottom slice and generally increasing values for higher located slices can be noticed. Lower values of diffusion coefficients for the bottom slice may be caused by the gravitational fall of cells and their squeezing due to the higher pressure exerted by the slices located above them. The decrease of *D*_2_ cannot be counteracted by the partially or to a greater extent damaged internal structure of cells.

Mean molar fractions *f*_1_ and *f*_2_ of *D*_1_ and *D*_2_, respectively, changed after 6 days of incubation (Table 2). Mean *f*_1_ decreased from 0.97 to 0.8 with a mean *D*_1_ drop from 1.21 × 10^−9^ m^2^ s^−1^ to 0.61 × 10^−9^ m^2^ s^−1^, while *f*_2_ increased from 0.0725 to 0.2 with no change of a mean *D*_2_. If no cellular death and only cell condensation occurred, *f*_1_ would be unchanged in favour of *f*_2_, only *D*_1_ would change (higher cells fraction with significantly smaller *D*). Therefore, the most possible scenario is that some part of the cells died in the process of necrosis or apoptosis. The part of apoptotic cells would release apoptotic bodies with membrane‐enveloped DNA fragments. They would have similar properties to the nucleus and collocate with a nuclear signal, giving a higher molar fraction of a *D*_2_ component. Both cell death mechanisms result in DNA release and medium coagulation leading to the decrease of *D*_1_ diffusion coefficient. Hence, it is suspected that *f*_2_ increase was mostly due to apoptosis, while *D*_1_ decrease was associated with the rise of density and viscosity resulting from DNA issued from cells destroyed by necrosis and partly by apoptosis.

### 4.4 Determination of MSCs’ self‐diffusion coefficient

In the previous work,^16^ it was proposed to use simulations of a time‐dependent diffusion coefficient (TDDC) for the diffusion times applied in the real experiments to identify cellular compartments. Experimental TDDC is associated with a given cellular structure if it corresponds with the simulated one for this structure (details of simulations used in this study are presented in Section S1). However, simulations require prior knowledge on the free (ie for *t*_d_ →0) self‐diffusion coefficient, *D*_0_, associated with a given water pool. Hence, simulations for MSCs are quite inconvenient due to the lack of information about in‐cell *D*_0_s. This is in a total contrast to other cells like yeast, which are well‐characterized in the literature. Therefore, a slightly different pattern was applied for the estimation of intracellular self‐diffusion coefficients.

Firstly, this analysis derives from experiments conducted in a constant time‐steady gradient, from which the nuclear size and self‐diffusion coefficient were determined (see Section 4.). Considering that nuclear residence time, *τ*_nucl_, is significantly shorter than applied diffusion times,^16^ peak 3 (Figure 3E,F) originates from the effective diffusion coefficient of exchanging water cytoplasm and nucleus, *D*_intra_(*t*_d_) = *f*_nucl_·*D*_nucl_(*t*_d_) + *f*_cyto_·*D*_cyto_(*t*_d_), where *f*_nucl_, *f*_cyto_, *D*_nucl_ and *D*_cyto_ are molar fraction of nuclear water, molar fraction of cytoplasmic water, diffusion coefficient in the nucleus and diffusion coefficient cytoplasm, respectively.

It is important that for *t*_d_ < *τ*_nucl_, signal attenuation due to diffusion in nucleus would be so small that would require a very high signal‐to‐noise ratio to be distinguished as a separate component, especially in small samples for which *f*_nucl_ is low (in this study it is equal to ~0.24%–1.5% of a total signal). Practically, if pulsed‐field gradient (PFG) techniques are used, most of the attenuation will come from diffusion in cytoplasm. Therefore, for *t*_d_ ≫ *τ*_nucl_ effective/intracellular diffusion coefficients *D*_intra_ will be observed, while for *t*_d_→0 *D*_0,intra_~*D*_0,cyto_. Based on the fact that intracellular self‐diffusion coefficients of 0.68 × 10^−9^ m^2^s^−1^ ^25^ and 0.65 × 10^−9^ m^2^ s^−1^ ^26^ were obtained for yeast cells, while self‐diffusion coefficient of 0.69 × 10^−9^ m^2^ s^−1^ ^16^ was obtained for yeast's cytoplasm, the *D*_0,intra_~*D*_0,cyto_ approximation seems to be justified for PFG in moderate gradient strengths. Since self‐diffusion coefficient of nucleus is known, *D*_cyto_(*t*_d_) was extracted from *D*_intra_(*t*_d_) and used for determination of *D*_0,cyto_. Estimated *D*_0,cyto_ was equal to 0.22 × 10^−9^ m^2^ s^−1^ and 0.29 × 10^−9^ m^2^ s^−1^ for *D*_cyto_(*t*_d_) = 0.165 × 10^−9^ m^2^ s^−1^ and 0.219 × 10^−9^ m^2^ s^−1^, respectively (for details of estimation of *D*_0,cyto_ required for simulations see Section S2).

#### 4.4.1 Verification of *D*_0,cyto_ by comparison with simulated TDDCs

For several theoretical *D*_0,cyto_s from the range of 0.2 × 10^−9^ m^2^ s^−1^ to 1.0 × 10^−9^ m^2^ s^−1^, TDDCs were simulated and experimental *D*_cyto_(*t*_d_) were compared with them. In Figure S1C, it can be seen that experimental *D*_cyto_(*t*_d_) lie close to the simulated TDDC assuming *D*_0,cyto_ = 0.22 × 10^−9^ m^2^ s^−1^ and 0.29 × 10^−9^ m^2^ s^−1^ for *D*_cyto_(*t*_d_) = 0.165 × 10^−9^ m^2^ s^−1^ and 0.219 × 10^−9^ m^2^ s^−1^, respectively. Based on Figure S1C, it can be concluded that in the restricting geometry with the diameter of *d* = 15 μm, for *D*_0_ ≤ 0.8 × 10^−9^ m^2^ s^−1^ molecules are in the free diffusion regime in the range of *t*_d_ = 0.1–50 ms, where Mitra's relation is valid. Therefore, more reliable *D*_0,cyto_ *~D*_0,intra_ can be estimated from Mitra's formula and is equal to 0.205 × 10^−9^ m^2^ s^−1^ and 0.283 × 10^−9^ m^2^ s^−1^ for *D*_cyto_(*t*_d_) = 0.165 × 10^−9^ m^2^ s^−1^ and 0.219 × 10^−9^ m^2^ s^−1^, respectively.

Taking into consideration that *D*_cyto_(*t*_d_) = 0.219 × 10^−9^ m^2^ s^−1^ results from the higher cells concentration, it can be suspected that ILT was more accurate in comparison with the three times lower concentration and the real *D*_0,cyto_ can be assumed to be equal to ~0.283 × 10^−9^ m^2^ s^−1^. This value is in the range of 0.15–0.63 × 10^−9^ m^2^ s^−1^ obtained by Tanner for the intracellular self‐diffusion coefficient of different cells in vitro.^27^ Such small diffusivity indicates rather high cytoplasmic viscosity. For example, ~3 times higher viscosity of blood compared with water at 37 °C results in an about two to three times smaller diffusivity (0.9–1.65 × 10^−9^ m^2^ s^−1^ ^28^). Taking into consideration that the viscosity of MSCs at 20°C is equal to ~2.71 Pa s,^29^ *D*_0,cyto_ of MSCs suggests that their cytoplasm contains either higher dry weight (resulting in a considerable *D*_0_ reduction), ions (brine shrimp cells in the work of Tanner^27^ had *D*_0_ = 0.35 × 10^−9^ m^2^ s^−1^) or lipids (*D*_0_ = 0.015 × 10^−9^ m^2^ s^−1^ ^27^).

### 4.5 New insights in the context of current MRI applications for the investigation of MSCs

Despite the ongoing investigations concerning MSCs (eg differentiation, viability, ontogenesis), attempts at the clinical applications of MSCs, especially for the civilization‐driven conditions, are boosted. Over the years, many studies on the application of MRI to MSCs monitoring have been reported. Most of them were oriented towards in vivo experiments, mainly related to the characterization of treatment effects^30^, ^31^ or tracking MRI‐labelled cells.^32^, ^33^, ^34^ Treatment effects are usually evaluated through the change of volume or size of a given region (eg tumour, infarct and cartilage) on MR images or *T*_1_, *T*_2_ and apparent diffusion coefficient (ADC) mapping. MRI‐labelled cells are tracked by shortened *T*_2_ or *T*_2_^*^ values, which result from the uptake of nanoparticles by the MSCs.

From the point of view of this study, tracking and differentiation of MSCs are particularly meaningful. As mentioned, MRI usage for this purpose is inextricably connected to the application of contrast agents (CAs), such as iron‐oxide or gadolinium‐based. The role of CAs is to change MRI‐derived parameters (*T*_1_, *T*_2_, ADC) in order to differentiate MSCs from the tissue. The meaning of the characterization of biophysical properties of MSCs for their distinguishing from primary, cancer and differentiated cells was pointed out.^35^, ^36^ Through our approach, we provide complementary parameters obtained non‐invasively for MSCs *in vitro*. The characterization of MSCs by means of 1D and 2D relaxometry revealed several MSCs‐specific features, including diffusional and relaxational behaviour. First of all, MSCs are characterized by a significantly smaller intracellular self‐diffusion coefficient, *D*_0,intra_ = 0.283 × 10^−9^ m^2^ s^−1^, in comparison with many other human cell types. For example, a diffusion coefficient of 0.45 × 10^−9^ m^2^ s^−1^ was reported for astrocytes, 1.06 × 10^−9^ m^2^ s^−1^ for cardiomyocytes,^37^ 0.9–1.6 × 10^−9^ m^2^ s^−1^ for axons measured longitudinally, while 0.3–0.5 × 10^−9^ m^2^ s^−1^ for axons measured perpendicularly,^38^ ~1 × 10^−9^ m^2^ s^−1^ for glia,^39^ 1.38 × 10^−9^ m^2^ s^−1^ for chondrocytes,^40^ ~0.8 × 10^−9^ m^2^ s^−1^ for white matter and ~1.2 × 10^−9^ m^2^ s^−1^ for grey matter.^41^ This gives potential to differentiate MSCs in these tissues. As shown in Sections 2 and 3, such a value strongly influences not only the effective diffusion coefficient in the layer of MSCs but also in the volume of suspended cells. Therefore, it seems that diffusion can be used as a potential biomarker for tracking MSCs non‐invasively, without the necessity of using CAs to change the intracellular properties of the cells.

## 5 SUMMARY

The study revealed the capability of a low field system to detect signals from cells in the samples with a low concentration of cells in the suspensions or low amounts of the sample without any contrasting agents. To sum up, based on the results from 1D: *T*_1_, *T*_2,_ *D*, 2D: *T*_1_‐*T*_2,_ *D*‐*T*_2_ measurements it was possible to:

- determine specific parameters for WJMSC of *T*_2_ *relaxation times*, *T*_2_ = 118–350 ms, and *diffusion coefficients D*_intra_ = 0.0163–0.0216 × 10^−9^ m^2^ s^−1^ and *D*_extra_ = 0.93–1.45 × 10^−9^ m^2^ s^−1^ corresponding to intra‐ and extracellular water pools, respectively;
- estimate *MSCs’ size* equal to ~15 μm from *D*‐*T*_2_ measurements;
- assess the effective diffusion coefficient for a *single layer of MSCs* cultured in a Petri dish, *D*_eff_ = 1.69 × 10^−9^ m^2^ s^−1^ allowing the determination of cells fraction (~28%);
- find evidence of *diffusion in the in‐cell structures* associated mainly with the nucleus characterized by *D*_0,nucl_ = 0.095 × 10^−9^ m^2^ s^−1^ and radius *R*_nucl_ = 2 μm;
- determine cell *viability* reflected in the effective diffusion coefficients (*D*_eff,1_ = 0.46–0.78 × 10^−9^ m^2^ s^−1^) reflecting apoptosis and necrosis of cells after 6 days of incubation;
- register *D*_eff_s indicating changed physical properties of the suspension due to *cell destruction* and the increase of *DNA‐rich components* with properties similar to a nucleus;
- estimate *MSCs’ intracellular self‐diffusion coefficient*, *D*_0,intra_ = 0.283 × 10^−9^ m^2^ s^−1^.

In further research, the known specific NMR parameters will be used to estimate the location of stem cells in organs undergoing therapy in MRI diagnosis *in vivo*, as well as to learn about their quantitative and qualitative characteristics in *in vitro* suspensions. A very important issue that has to be addressed in the further, research on our method is the possibility of distinguishing different fractions of cells in their mixture in an *in vitro* experiment. Another challenge will be to try to distinguish MSCs from other cell types *in vivo* through clinical MRI imaging.

## 6 CONCLUSIONS

The determination of specific parameters for MSCs in LF‐NMR opens up the possibility of research on the detection of these cells *in vivo* as well as attempts at the determination of their quantity or vitality in the source tissues, such as the umbilical cord, in *in vitro* studies. The application of a single‐sided NMR device with a strong magnetic field gradient allowed the attainment of very thin slices and the detection of a single cell layer in the Petri dish. This introduces the possibility of examination of MSCs properties and their differences at the individual cell layer. The cells setting in the Petri dish also has the advantage of imitating the *in vivo* environment. It relies on the presence of a limited number of cells in the watery ambience, similarly to the case of cells reposition on the tissue. In this way, the character of diffusivity change can reflect the presence and amount of MSCs.

Experiments in the cylindrical container enabled studying cell viability through the change of the diffusion coefficients and components’ fraction. In order to determine the MSCs lifetime, the viability curve has to be examined. The two surveys carried out within a six‐day interval were aimed at tracking any evidence of cell death by the change of diffusivities, something which was accomplished. This indicates that diffusion can be proposed as a natural biomarker of a cell viability. Based on the obtained results, it seems that necrosis and apoptosis can be distinguished, which can be achieved thanks to the ability of NMR‐MoUSE device to detect low diffusivity components, similar to a nucleus. This provides the opportunity to trace tissue destruction or tissue remodelling through the evidence of elements of cell dissolution. However, reference studies, such as microscopy, are required.

## CONFLICT OF INTEREST

The authors declare that they have no competing interests.

## AUTHOR CONTRIBUTIONS

**Artur T. Krzyżak:** Conceptualization (lead); data curation (equal); funding acquisition (equal); investigation (equal); methodology (equal); project administration (equal); software (equal); supervision (lead); validation (equal); writing – original draft (equal); writing – review and editing (equal). **Iwona Habina‐Skrzyniarz:** Data curation (equal); formal analysis (equal); investigation (equal); software (equal); visualization (equal); writing – original draft (equal). **Weronika Mazur:** Conceptualization (supporting); data curation (equal); formal analysis (equal); software (equal); validation (equal); visualization (equal); writing – original draft (equal); writing – review and editing (equal). **Maciej Sułkowski:** Data curation (supporting); investigation (supporting); resources (equal); visualization (supporting); writing – original draft (supporting). **Marta Kot:** Data curation (supporting); investigation (supporting); resources (supporting); visualization (supporting); Writing – original draft (supporting). **Marcin Majka:** Conceptualization (equal); funding acquisition (equal); methodology (equal); project administration (equal); resources (equal); supervision (equal); validation (equal); writing – review and editing (equal).

## Supporting information

Supplementary Material

Fig S1

## Data Availability

The data that support the findings of this study are openly available in Mendeley Data repository at http://dx.doi.org/10.17632/n3sfpcyp4z.1. Simulations code supporting the current study have not been deposited in a public repository, because it is similar to the widely used and commonly available variants of random walk approach for diffusion simulations (The MathWorks Inc.), but are available from the corresponding author on request.
