# Supplementary material for: Nuclear magnetic resonance footprint of Wharton Jelly mesenchymal stem cells death mechanisms and distinctive in‐cell biophysical properties in vitro
Source: J Cell Mol Med. 2022 Jan 25;26(5):1501–14. doi: 10.1111/jcmm.17178 (PMC8899161; doi:10.1111/jcmm.17178)
Supplement: Supplementary file 1 — Supplementary Material [file JCMM-26-1501-s002.docx]

**Nuclear magnetic resonance footprint of Wharton Jelly mesenchymal stem cells death mechanisms and distinctive in-cell biophysical properties *in vitro***

*Artur T. Krzyżak^a,^*, Iwona Habina-Skrzyniarz^a^, Weronika Mazur^a,b^, Maciej Sułkowski^c^, Marta Kot^c^, Marcin Majka^c^*

^a^Faculty of Geology, Geophysics and Environmental Protection, AGH University of Science and Technology, al. Mickiewicza 30, 30-059 Cracow, Poland

^b^Faculty of Physics and Applied Computer Science, AGH University of Science and Technology, al. Mickiewicza 30, 30-059 Cracow, Poland

^c^Jagiellonian University Collegium Medicum, Department of Transplantation, ul. Wielicka 265, 30-663 Cracow, Poland

***Corresponding author**; e-mail: [akrzyzak@agh.edu.pl](mailto:akrzyzak@agh.edu.pl), Telephone: +48 12 617 52 91, Address: Faculty of Geology, Geophysics and Environmental Protection, AGH University of Science and Technology, al. Mickiewicza 30, 30-059 Cracow, Poland

**Supporting Information**

**S1. Random walk simulations**

The origin of the peaks from the *D-T_2_* maps was verified by the comparison of experimental and theoretical diffusion coefficients. The latter were determined from the Mitra relation ^1^ and the Einstein-Smoluchowski diffusion equation ^2^ by using the root mean square displacement of water molecules in cellular microgeometry. This was achieved by using the mean displacement of random walkers obtained from Monte Carlo simulations conducted analogously to the previous work ^3^ in MATLAB (R2019b) (The MathWorks Inc., Natick, MA, USA). 2D non-exchangeable microgeometry was assumed with sizes of cells and nuclei analogous to those determined based on the experiments, particle jump duration was equal to *t_s_*=5 μs, step length, *σ*, was dependent on the self-diffusion coefficient, *D_0_* m^2^ s^-1^, *σ^2^*=4*D_0_t_s_*, while the number of random walk steps depended on diffusion time, *t_d_*=*Δ – δ*/3, based on the relation *N*=*t_d_*/*t_s_*. Apparent diffusion coefficients in simulations were determined from the average of root mean square displacements of 5000 random walkers.

**S2. Estimation of MSCs’ intracellular D_0_ required for simulations**

It was certain that *D_0,cyto_* is in the range from the experimental diffusion coefficient, *D_cyto_*(*t_d_*), to the buffer’s diffusion coefficient (D of peak 1 in Figure 3A), *D_buffer_*. Firstly, characteristic lengths were estimated assuming *D_0,cyto_*=*D_cyto_*(*t_d_*) and *D_0,cyto_*=*D_buffer_* in order to identify diffusion regime for water in the cell. There are three characteristic lengths: restriction length, *l_s_*=*d*, diffusion length, $l_{d}=\sqrt{D_{0}t_{d}}$, and dephasing length, $l_{g}=\left( \frac{D_{0}}{\gamma G} \right)^{1/3}$. Molecules can be in the one of the three diffusion regimes: free diffusion (F), localization regime (L) or motional averaging (M), if the shortest length is *l_d_*, *l_g_* or *l_s_*, respectively. In each of these regimes TDDC is described by a different formula. For example, for the regimes of interest, diffusion coefficient in free diffusion and localization regimes can be determined from Mitra’s and Einstein-Smoluchowski equations, respectively. Calculations showed that for *D_0,cyto_*=*D_buffer_* water is in the localization regime for all of the gradient strengths. For *D_0,cyto_*=*D_intra_*(*t_d_*) intracellular water molecules are in the free diffusion regime for low gradient strengths (e.g. arbitrarily chosen *G*=0.1 T m^-1^), while comes into the localization regime for *G_max_*. Therefore, it was reasonable to estimate *D_0,cyto_* from the Padé approximant (interpolation between to functions) assuming α and θ parameters based on the work of Latour et al. ^4^ *D_0,cyto_* was equal to 2.2×10^-10^ m^2^ s^-1^ and 2.9×10^-10^ m^2^ s^-1^ for suspension with cells concentration of 5 mln/0.5 ml and 15 mln/0.5 ml, respectively. These values were baselines for choosing *D_0,cyto_*s.

**References**

1. Mitra PP, Sen MPN, Schwartz LM, Doussal P Le. Diffusion Propagator as a Probe of the Structure of Porous Media. *Phys Rev Lett*. 1992;68(24):3555-3558.

2. Islam MA. Einstein–Smoluchowski Diffusion Equation: A Discussion. *Phys Scr*. 2004;70(2-3):120-125. doi:10.1088/0031-8949/70/2-3/008

3. Mazur W, Krzyżak AT. Attempts at the Characterization of In-Cell Biophysical Processes Non-Invasively — Quantitative. *Cells*. 2020;9(9):2124. doi:10.3390/cells9092124

4. Latour LL, Mitra PP, Kleinberg RL, Sotak CH. Time-Dependent Diffusion Coefficient of Fluids in Porous Media as a Probe of Surface-to-Volume Ratio. *J Magn Reson*. 1993;101(3):342-346. doi:https://doi.org/10.1006/jmra.1993.1056
