# Supplementary figures and images for: Nuclear magnetic resonance footprint of Wharton Jelly mesenchymal stem cells death mechanisms and distinctive in‐cell biophysical properties in vitro
Source: J Cell Mol Med. 2022 Jan 25;26(5):1501–14. doi: 10.1111/jcmm.17178 (PMC8899161; doi:10.1111/jcmm.17178)

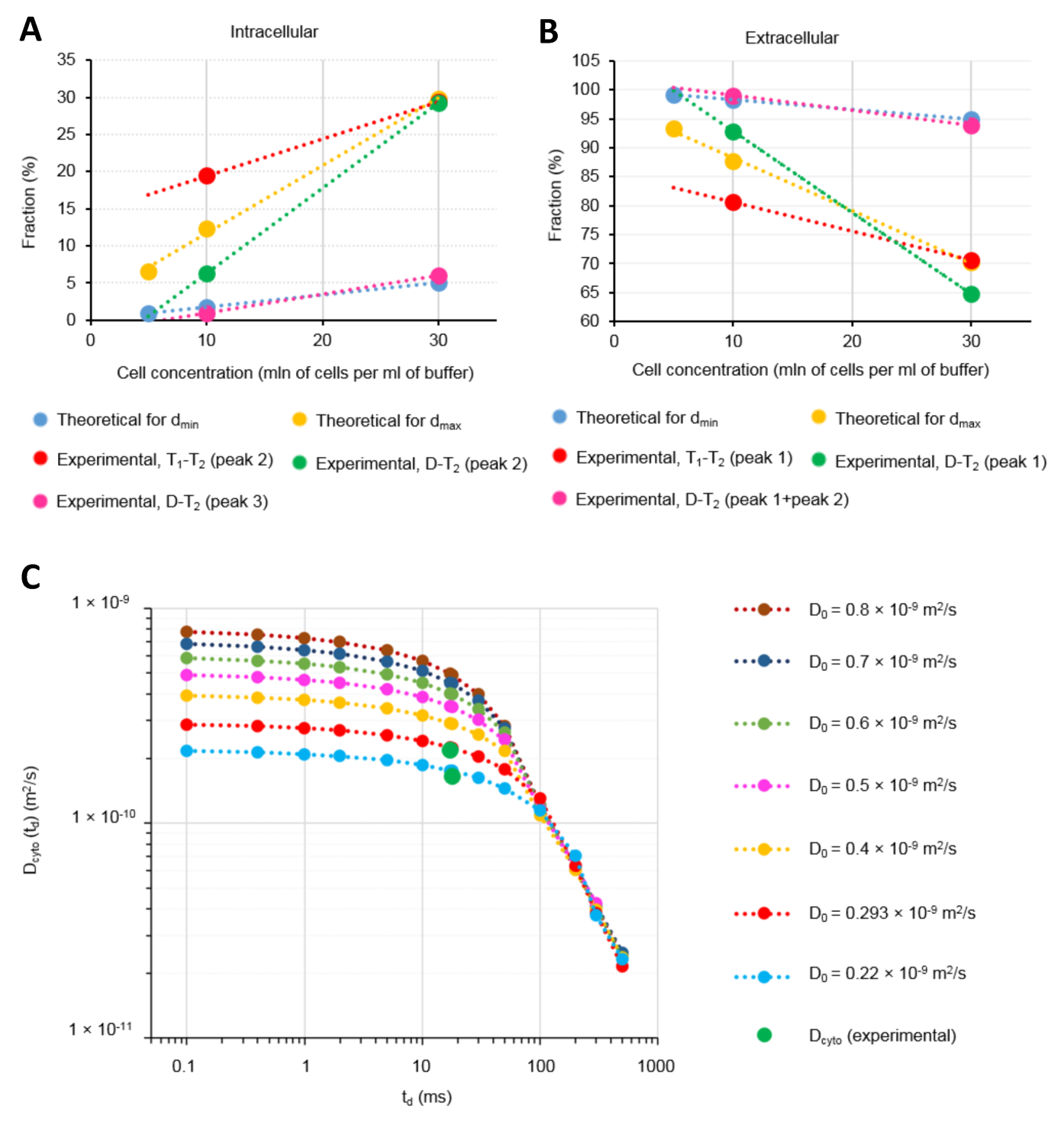

Supplement: Supplementary file 2 — Fig S1 [file JCMM-26-1501-s001.tif]
